# Molecular Evidence of Zoonotic Pathogens in Free-Living Wild Birds: A Greek Surveillance Study

**DOI:** 10.3390/pathogens15030308

**Published:** 2026-03-11

**Authors:** Sokratis Perdikaris, Maria Evangelidou, George Diamantopoulos, Evangelia Kofidou, Grigorios Markakis, Dimitrios Vourvidis, Emmanouil Papadogiannakis, Anastasia Komnenou, Emmanouil Angelakis

**Affiliations:** 1 Diagnostic Department and Public Health Laboratories, Hellenic Pasteur Institute, 11521 Athens, Greece; 2 Department of Public Health Policy, School of Public Health, University of West Attica, 11521 Athens, Greece; 3 Directorate of Animal Health, General Directorate of Veterinary Services, Ministry of Rural Development and Food, 10438 Athens, Greece; 4 School of Veterinary Medicine, Faculty of Health Sciences, Aristotle University of Thessaloniki, 54124 Thessaloniki, Greece; 5 ANIMA-Hellenic Wildlife Rehabilitation Center, 17676 Athens, Greece; 6 Directorate of Veterinary Laboratory Center, General Directorate of Veterinary Services, Ministry of Rural Development and Food, 15341 Athens, Greece

**Keywords:** one health, zoonotic pathogens, wild birds, Greece, molecular detection, *Cryptococcus*, *Chlamydia psittaci*, *Giardia duodenalis*, *Cryptosporidium*, *Mycobacterium avium*

## Abstract

Wild birds are increasingly recognized as contributors to the circulation and environmental dissemination of zoonotic pathogens, yet data from Greece remain limited, particularly for raptors, corvids, and water birds. This study investigated selected parasitic, mycotic, and bacterial pathogens of public health relevance in free-living wild birds originating from various regions of Greece and admitted to two animal health care facilities. Between November 2023 and May 2025, cloacal swabs from 212 injured or sick birds were analyzed using quantitative PCR for *Cryptococcus* spp., *Chlamydia psittaci*, *Giardia duodenalis*, *Cryptosporidium* spp., and *Mycobacterium avium*. At least one pathogen was detected in 37 samples (17.5%), with *Cryptococcus* spp. being the most frequent agent (11.8%), followed by *C. psittaci* (3.8%), *G. duodenalis* (0.9%), and *Cryptosporidium* spp. (0.9%). *M. avium* was not detected. Pathogen occurrence varied by bird category, genus, and region, and was associated with health status. To the best of our knowledge, this study provides the first molecular evidence worldwide of *G. duodenalis* in *Ardea* spp., *Cryptosporidium* spp. in *Asio otus*, and *C. psittaci* in *Botaurus stellaris* and *Plegadis falcinellus*. These findings highlight wild birds as potential zoonotic reservoirs and support the implementation of One Health–oriented surveillance programs.

## 1. Introduction

Zoonoses have been documented and affected human populations since antiquity. It is estimated that 61% of all known human pathogens are of zoonotic origin, and the same applies to 75% of pathogens associated with emerging infectious diseases in humans [1]. Emerging zoonoses have been occurring at increasingly high rates since 1940 [2], exerting substantial impacts on public health and causing significant socio-economic consequences. The importance of wildlife as a reservoir and source of zoonotic transmission to humans is well established and widely recognized by the scientific community and relevant international organizations (WHO, FAO, WOAH). This significance becomes even more apparent when considering that 71.8% of emerging zoonotic diseases originate from wildlife species [2].

Wild birds are globally recognized as important reservoirs and disseminators of a wide array of infectious agents (parasites, fungi, bacteria, and viruses), including pathogens of significant zoonotic relevance. Their ability for long-distance migration, ecological adaptability, and frequent use of human-modified environments facilitates the circulation and spread of microorganisms across continents and between wildlife, domestic animals, and humans [3]. Investigating their occurrence in wild avian populations is therefore essential for identifying potential sources of environmental contamination, assessing risks to animal and human health, and shaping One Health-oriented surveillance and monitoring strategies.

Among the zoonoses associated with wild birds, cryptococcosis, cryptosporidiosis, giardiasis, avian chlamydiosis, and avian tuberculosis are of particular relevance [4,5]. Despite their significance, data on these zoonotic diseases in free-living wild birds remain limited in Greece, particularly for birds of prey, aquatic birds, and corvids. To date, only the presence of *Cryptosporidium* spp. and *Giardia* spp. at the genus level has been confirmed in ten and four bird species, respectively, belonging to raptors and water birds, based on samples collected primarily from the northern regions of the country [6].

The list of wild birds in Greece includes 442 species, of which approximately 60% are migratory, occurring seasonally as summer breeders, winter visitors, passage migrants, or non-breeding visitors [7]. This high proportion of migratory species facilitates the seasonal introduction and circulation of pathogens, which can ultimately increase the risk of zoonotic spillover events. A large number of individuals are admitted annually to wildlife care facilities (veterinary university clinics, rehabilitation centers, first-aid stations) for various reasons, such as accidents, orphaning, and disease. These establishments provide unique opportunities to investigate zoonotic pathogens in wildlife, facilitating sample acquisition and laboratory testing from populations that would be difficult to examine under natural conditions. Furthermore, the research value of such facilities is underscored by the fact that birds admitted for treatment often experience immunosuppression due to stress, thereby increasing the risk of pathogen shedding [3].

Although fungal zoonoses of wildlife have traditionally received limited attention, their prevalence in the human population has increased markedly over recent decades, with the most severe impacts being observed in immunocompromised individuals. Members of the *Cryptococcus neoformans* and *Cryptococcus gattii* species complexes are the principal etiological agents of cryptococcal meningitis, which is estimated to cause approximately 181,000 deaths globally each year, predominantly among people living with HIV/AIDS [8]. Therefore, it is essential to highlight and systematically investigate such mycoses in wildlife populations, particularly birds, which can carry *Cryptococcus* species and contribute to environmental dissemination, as already demonstrated in nearby Italy [9,10,11,12].

Protozoan parasites of the genera *Cryptosporidium* and *Giardia*, including zoonotic species, have been frequently detected in wild birds worldwide, with representative reports from the USA (North America), China (Asia), Australia (Oceania), and Spain (Europe) [13,14,15]. *Cryptosporidium* species infect a broad range of vertebrates and are major causes of diarrheal disease in humans, with *C. parvum* and *C. hominis* accounting for approximately 95% of cases [16]. Transmission occurs primarily via the fecal–oral route through contaminated water or food, as well as through direct contact with infected humans or animals and contaminated environments [17]. Giardiasis in humans is an infection caused by the protozoan *Giardia duodenalis* (syn. *G. intestinalis*, *G. lamblia*), especially assemblages A and B [16], with transmission occurring via the same fecal–oral pathways described for cryptosporidiosis.

Wild birds are also susceptible to various bacterial infections, including those belonging to the genera of *Chlamydia* and *Mycobacterium*. *Chlamydia psittaci*, the pathogen responsible for psittacosis in humans, is one of the best-known zoonotic bacteria associated with birds. It has been detected in approximately 465 bird species, causing clinical signs that vary, while bird infections may also be asymptomatic [18]. As in birds, the clinical manifestation in humans is variable, and in some cases, the disease may progress to pneumonia. It is estimated that about 1% of community-acquired pneumonia cases are attributable to *C. psittaci* infection [19]. The presence of the bacterium has been confirmed in numerous wild bird species, in at least 38 countries in all continents (e.g., Switzerland, Germany, Chile, USA, New Zealand, India, South Africa), but further research is recommended for specific groups such as raptors and water birds, which have received less attention compared to psittacines and pigeons [20]. Infections by mycobacteria of the *Mycobacterium avium* complex, most commonly *Mycobacterium avium* subsp. *avium*, are relatively common in farmed poultry or other captive birds. However, cases in wild birds, specifically in free-living raptor species, have also been reported, as in Majorca, Spain [21]. Wild birds are considered reservoirs and sources of dissemination of these mycobacteria through their droppings, thereby exposing other birds, animals, humans, and the environment, and facilitating transmission over prolonged periods [22]. In humans, mycobacteria of the *Mycobacterium avium* complex are classified as nontuberculous mycobacteria and primarily affect individuals with underlying respiratory conditions or immunosuppression.

Bird secretions and excretions, including feces, urine, and respiratory discharges, can harbor various pathogens that contaminate soil and water, thus contributing to pathogen spreading and disease transmission, with fecal shedding documented for each of the pathogens discussed above. Nevertheless, non-invasive sampling minimizes the risk of contamination associated with feces samples, making cloacal swabs an appropriate clinical specimen for monitoring the microbial burden in birds and the ecosystem.

Consequently, this study aims to investigate for the first time the presence of specific parasitic, mycotic, and bacterial pathogens in the cloacal swabs of certain groups of free-living wild birds from different areas across Greece. The data presented here will provide crucial information on the microbial load in wild bird populations and enhance zoonotic disease awareness. The data on the geographical distribution of detected pathogens, as well as the avian species exhibiting higher pathogen incidence, will be valuable for both epidemiologists and ornithologists involved in One Health-oriented surveillance and monitoring programs.

## 2. Materials and Methods

### 2.1. Bird Population and Sample Collection

During the period from November 2023 to May 2025, cloacal swabs were collected from a total of 212 injured or sick wild birds admitted for treatment to the “Exotic and Wildlife Unit” of the School of Veterinary Medicine at Aristotle University of Thessaloniki, and to the “Wildlife Rehabilitation Center-ANIMA”, a facility which has been officially approved for this purpose in accordance with the provisions laid down in national legislation. The animals were detected and captured across different regions throughout the country. They belonged to specific wild bird categories, namely raptors, corvids, and water birds, including seabirds and shorebirds.

To ensure the traceability of both birds and corresponding samples during all stages of rehabilitation and testing, a unique identifier number was assigned to each animal. The identifier numbers mentioned, along with the detailed information such as life stage, sex, location of discovery, reason for admission, and diagnosis, were individually recorded in an electronic database (data not provided).

A disposable, sterile specimen collection swab with tube was used to obtain the samples after the stabilization of the animals and prior to any systemic administration of antimicrobial drugs. Following their acquisition, the samples were directly stored on-site at a temperature of −20 °C. They were transferred, within a short time frame not exceeding 24 h, to be stored at −80 °C until further analysis.

### 2.2. Nucleic Acid Extraction

Nucleic acid extraction was performed on all 212 cloacal swabs after they were immersed in 1 mL PBS (Gly) using the QIAamp Power Fecal Pro DNA kit (Cat no. 51804, Qiagen, Hilden, Germany), optimized as follows. Briefly, 250 μL of the immersed cloacal swabs were added to 400 μL of the CD1 buffer, together with glass beads and 5 μL of SPUD as an external control for the presence of PCR inhibitors [23]. The samples were vortexed and then subjected to a FastPrep tissue homogenizer at maximum speed for 40 s. Following a 100 °C incubation for 10 min, 400 μL of the samples were transferred to a 2.0 mL-fresh, sterile microcentrifuge tube and were then subjected to an overnight incubation with 20 μL Proteinase K at 56 °C. Finally, 200 μL of the CD2 buffer was added to a 2.0 mL-fresh, sterile microcentrifuge tube in which 400 μL of the sample was transferred, and nucleic acid extraction was subsequently done according to the manufacturer’s protocol. After the nucleic acid extraction, all samples were quantified using a spectrophotometer to assess the quantity and purity of the nucleic acid yielded. All extracted nucleic acids were stored at −80 °C until further analysis.

### 2.3. PCR Amplification

The extracted nucleic acids were subjected to qPCR amplification using specific primers and probe sequences designed to target conserved regions of genes to detect the presence of *Cryptococcus* spp., *Chlamydia psittaci*, *Giardia duodenalis*, *Mycobacterium avium*, and *Cryptosporidium* spp. (Table 1). Each PCR amplification included both negative (showing no amplification) and positive controls, with the latter being specific to the target pathogens (*C. neoformans* for *Cryptococcus* spp. and *C. parvum* for *Cryptosporidium* spp.). All PCR reactions were run in duplicates. The Level of Detection (LOD) of the qPCR assays is reported in the Supplementary Table S1. Furthermore, a parallel qPCR experiment was run to exclude the presence of PCR inhibitors in the nucleic acid extracts using primers and a probe that target the specific external sequence, as previously described [23].

### 2.4. Statistical Analysis

Fisher’s Exact Test (expected cell counts < 5) was conducted to assess significant differences between positive and negative samples in different groups based on the criteria of age (adult vs. young), sex (male vs. female), and health status (injured vs. sick). Furthermore, for each bird category, the number of positive and negative samples per pathogen was compared to the corresponding numbers in the rest of the bird population (e.g., corvid vs. non-corvid *Chlamydia psittaci* positive vs. negative samples). All analyses were performed using GraphPad Prism software, version 6.0 (GraphPad Software, Inc., San Diego, CA, USA). Prevalence estimates are reported with 95% confidence intervals, and a *p*-value < 0.05 was considered statistically significant.

### 2.5. Ethics Approval Statement

For the purposes of this study, all the rules and criteria of ethics and bioethics governing the approval and operation of recognized, approved, and licensed by the State, Wildlife Rehabilitation Centers, and First Aid Stations, were applied in accordance with the legislative provisions of no. YPEN/DDD/88658/2929/2022 Joint Ministerial Decision of the Ministries of Environment, Energy, Rural Development, and Food. The cloacal swabs collected for the research purpose of this study came from wild birds that were temporarily housed in approved and licensed care centers that meet the technical, veterinary, and environmental criteria of the above decision, with the aim of their safe and sustainable rehabilitation into the natural environment.

## 3. Results

### 3.1. Bird Population and Geographical Distribution

During the period from November 2023 to May 2025, 212 birds’ cloacal swabs were tested for the presence of the pathogens mentioned above. The ratios of adult to young birds and males to females, where information was available, were approximately 3:1 and 2:1, respectively. The vast majority of the sampled birds, for which information was available, were admitted due to involvement in some kind of accident (157/166, 94.6%), and only nine of them (5.4%) were hospitalized due to illness.

**Figure 1:**
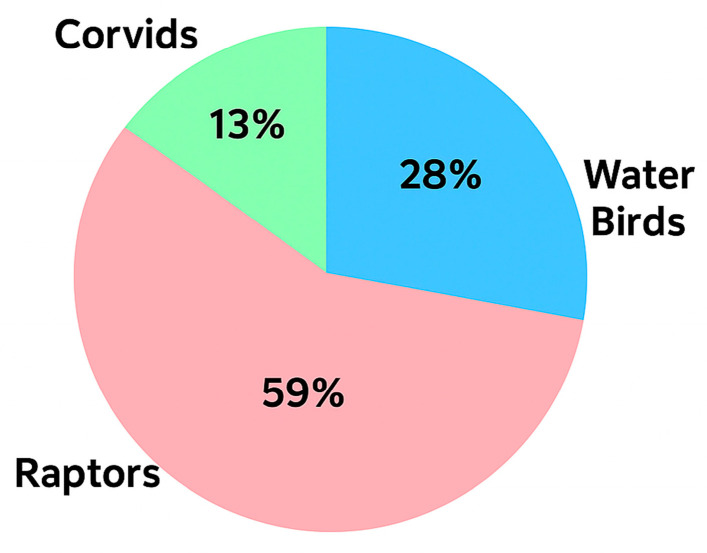
The wild bird groups that participated in this study and their corresponding percentages are illustrated in the pie chart.

The wild birds included in this study belong to three specific categories: raptors (125/212, 59%), corvids (27/212, 13%), and water birds (60/212, 28%), including seabirds and shorebirds (Figure 1), each one of them comprising a large number of different species. More specifically, 12, 3, and 11 different genera fall into the categories of raptors, corvids, and water birds, respectively. Detailed information on the genera and the corresponding numbers and percentages by which they are represented in the population of this study are shown in Table 2.

**Figure 2:**
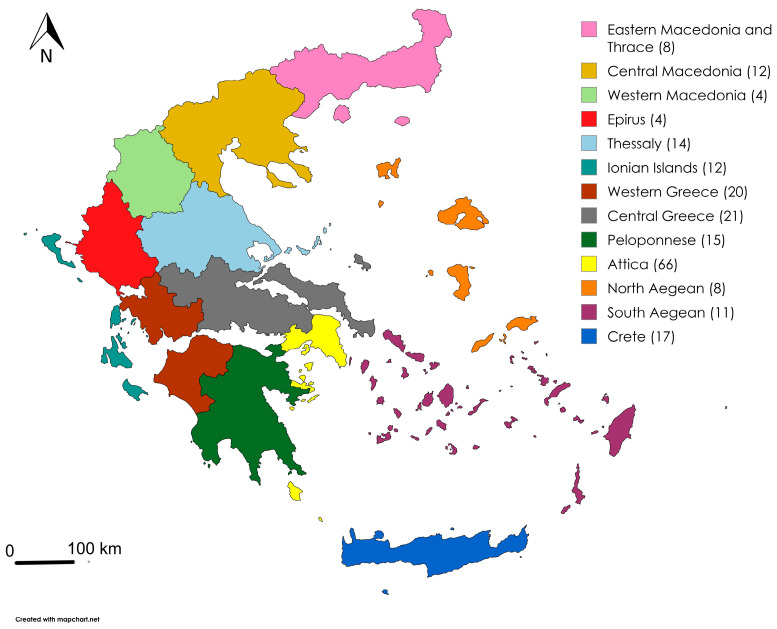
Geographical distribution of wild bird samples by NUTS 2 region in Greece, with numbers indicating samples collected from each region, https://www.mapchart.net/ (accessed on 2 December 2025).

Regarding their geographical distribution, the animals originated from different and representative areas of the country, as shown in Figure 2.

The geographical units and their corresponding proportions of the total samples and percentages are depicted in Table 3.

### 3.2. Pathogen Detection

Τhe extracted nucleic acids were subjected to qPCR amplification for the detection of the pathogens mentioned above. Thirty-seven samples (37/212, 17.5%) were found positive (Ct values ≤ 37) for one of the pathogens tested in this study. More specifically, 25/212 (11.8%) were found positive in *Cryptococcus* spp. (LOD 0.036 cp/μL), 8/212 (3.8%) in *Chlamydia psittaci* (LOD 0.9 cp/μL), 2/212 (0.9%) in *Giardia duodenalis* (LOD 1.2 cp/μL), and 2/212 (0.9%) in *Cryptosporidium* spp. (LOD 0.9 cp/μL). None of the samples identified positive for *Mycobacterium avium* (LOD 0.7 cp/μL).

The ratios of the positive samples between adult to young birds and males to females were 3:1 and 2:1, similar to the total population of samples (*p* = 0.644 and *p* = 1, respectively). In terms of admission, 24 (64.9%) and five (13.5%) out of 37 positive samples belong to the injured and the sick group, respectively (*p* = 0.0089), showing that the health status of the individuals can be associated with the occurrence of the pathogens tested here. Finally, swabbing dates of the positive samples are distributed equally in different periods around the year.

### 3.3. Pathogen Detection Among Different Categories and Genera

The presence of *Cryptococcus* was detected in all categories tested. More specifically, among the 25 positive samples for *Cryptococcus*, 14 (56%) belonged to the raptors, 10 (40%) to the water birds, and one (4%) to the corvids. Similarly, in terms of genus dominance, 7/14 (50%) of the *Cryptococcus*-positive raptor samples belonged to the genus *Buteo*, and 5/10 (50%) of the *Cryptococcus*-positive water bird samples belonged to the genus *Larus* (Table 4).

Among the eight *Chlamydia psittaci*-positive samples, five (62.5%) belonged to the corvids (*p* = 0.001 compared to the non-corvid group), two (25%) to the water birds, and one (12.5%) to the raptors. The genus *Pica* dominated the *C. psittaci* positive samples, accounting for the 80% (4/5) of the positive samples falling into the corresponding bird group (Table 4). Two samples were found positive for the presence of *Giardia duodenalis*, both belonging to the water birds and the genus *Ardea*. Finally, two samples were found positive for the presence of *Cryptosporidium* spp., both belonging to the category of raptors. The detailed information on the genera and species found positive in the pathogens tested in this study, their corresponding numbers per pathogen expressed as absolute numbers and percentages, together with the total prevalence for each species are shown in Table 4.

### 3.4. Geographical Distribution of the Positive-Tested Individuals

*Cryptococcus* was detected in samples from all different geographical units of the country except Epirus. Approximately half of the total *Cryptococcus* spp. positive samples originated from three geographical regions; Attica (5/25, 20%), Central Macedonia (4/25, 16%) and Central Greece (3/25, 12%). *C. psittaci* was found in five of the 13 geographical units of the country, and more than half of the total positive samples originated from Attica (3/8, 37.5%) and Central Greece (2/8, 25%). Finally, the two samples found positive in *G. duodenalis* originated from the Ionian Islands and Crete, and the two *Cryptosporidium* spp. positive samples located in Peloponnese and Attica. The detailed geographical distribution of the positive samples, together with the percentage that each geographical unit contributes to the total amount of the positive samples per pathogen, is shown in Table 5.

### 3.5. Geographical Burden Load and Genera-Based Positivity Rates

The geographical regions that were found to be burdened to a greater extent, based on the numbers of the positive samples proportional to the total numbers of samples from each geographical unit, are Central Macedonia (33.3%) for *Cryptococcus* spp., Central Greece (9.5%) for *C. psittaci*, Ionian Islands (8.3%) for *G. duodenalis* and Peloponnese (6.7%) for *Cryptosporidium* spp. Furthermore, different samples originating from the regions of the Ionian Islands, Attica, and Crete were found to be positive for three of the pathogens tested in this study. Collectively for all the pathogens tested here, the areas that were affected the most were Central (33.3%) and Western (25%) Macedonia, Ionian Islands (25%), and the North Aegean (25%) (Table 6).

A genus-based analysis of the data provided here showed that specific genera displayed higher positivity rates than others. More specifically, the category of the raptors consisted of 12 genera, six of which were found to carry the pathogens tested, with *Cryptococcus* being the most common one. Although the genus *Buteo* displays the highest absolute number in terms of sample positivity, the total positivity rate is 16.7% compared to the 100% positivity rate demonstrated in the genera *Circaetus*, *Pernis*, and *Strix*. The category of corvids consisted of three genera, two of which tested positive for the pathogens investigated in this study, with *C. psittaci* being the most abundant pathogen in this category. The genus with the highest positivity rate in this bird group is *Pica* (33%). Finally, eight of the 11 different genera that comprise the category of water birds were found positive in one of the pathogens of this study, exhibiting different positivity rates ranging from 15–100%. As with raptors, *Cryptococcus* again appears to be the most frequently detected pathogen. The detailed information on the genera and species found positive in the pathogens tested in this study, their corresponding numbers per pathogen expressed as absolute numbers and percentages, together with the total prevalence for each species are shown in Table 4.

## 4. Discussion

The aim of this study is to further heighten the role of wild avian populations as reservoirs and disseminators of a wide range of infectious agents, particularly in Greece. For that purpose, we focused on specific pathogens of public health relevance by investigating the occurrence of *Cryptococcus* spp., *Chlamydia psittaci*, *Giardia duodenalis*, *Cryptosporidium* spp., and *Mycobacterium avium* in cloacal swabs from numerous wild bird species from different areas across the country. Our results indicate that avian wildlife in Greece indeed represents an important mediator and spreader at least for the pathogens investigated in this study, since 17.5% of samples were positive in one of the pathogens tested here, except *Mycobacterium avium*.

Data presented here confirm a previous study showing that neither sex nor age of the bird population seems to pose a risk factor [28], although there are previous studies showing contradictory results [29,30]. Positive samples are distributed equally in different periods around the year, showing that seasonality does not affect the occurrence of the tested pathogens. On the contrary, the health status of the birds is associated with the pathogen prevalence, as the overall prevalence in this group is 56% compared to the 17.5% in the entire study group (*p* = 0.0089 when comparing positive samples between sick and injured birds).

Nevertheless, this study has potential limitations. First, the data presented here were obtained from wild birds admitted to rehabilitation centers due to injury or illness. Consequently, our dataset likely over-represents individuals with compromised health and may not fully reflect pathogen prevalence or diversity in the general wild bird population. While our findings provide valuable insights into pathogen occurrence in injured or sick wild birds, they should be interpreted as representative of this subset rather than of the broader free-living wild bird community. Second, the low number of positive samples for certain pathogens limited the statistical power to detect potential associations with host variables. Consequently, the findings of this study should be interpreted within the context of these constraints. Despite these limitations, the present study provides important preliminary insights into pathogen occurrence in this understudied host population.

*Cryptococcus* spp. was the most frequently detected pathogen among all pathogens identified in the bird population included in this study, accounting for 25 out of 37 cases (68%). Raptors and water birds showed the highest proportion of *Cryptococcus*-positive samples, with 11.2% (14/125) and 17% (10/60), respectively, compared to 3.7% in corvids. Among the affected birds, the genera *Buteo* and *Larus* accounted for nearly half (48%) of all *Cryptococcus*-positive samples, with the remaining 52% distributed across ten other genera. Although molecular detection of *Cryptococcus* spp. in this study cannot be resolved to the species level, the assay targets conserved regions compatible with members of the *C. neoformans/C. gattii* species complexes; therefore, the presence of these pathogenic taxa cannot be excluded. However, previous surveillance [31] of wild avifauna has demonstrated that non-*neoformans/gattii Cryptococcus* and related genera are more frequently recovered than the highly pathogenic complexes, suggesting that our findings may reflect a similar ecological pattern. In this two-year surveillance study in Southern France, *Larus* was also found to be the second most affected genus, after *Streptopelia*, which was not included in our study. Despite its limitations, this study shows for the first time not only the presence of *Cryptococcus* spp. in wild avian life in every part of the country but also denotes the area of Central Macedonia as the most affected area by this pathogen.

The second most often encountered pathogen is *Chlamydia psittaci*. Eight of the 37 (22%) total positive samples were found positive, with most of them (62.5%) being detected in the category of the corvids (*p* = 0.001 when comparing positive samples between corvid and non-corvid birds). Several studies have reported that the *C. psittaci* prevalence is highly variable depending on the bird species [32]. A meta-analysis study showed that the prevalence can be between 2.2–95.6%, depending on the orders of bird populations and also the diagnostic techniques used [33]. Nevertheless, a study from Switzerland shows that the corvids are more susceptible to *C. psittaci* infection compared to the raptors [34], a finding also confirmed in the present study, to a higher percentage, though, than the one reported here (18.5%, Table 4), probably due to a higher number of birds and samples in the Swiss investigation. This study is the second report in the last 12 years showing the presence of *C. psittaci* in the species *Pica pica* [35]. It provides the first data on *C. psittaci* prevalence in the Greek avian wildlife, mainly in the central and southern parts of the country, with positive cases detected in the groups of raptors, corvids, and water birds that are globally under-investigated compared to psittacines and pigeons. Notably, we detected *C. psittaci* in *Botaurus stellaris* and *Plegadis falcinellus*, in which this pathogen has not previously been reported.

Both positive samples for *G. duodenalis* originated from species of the genus *Ardea*, namely *Ardea ibis* and *Ardea cinerea*. In total, five individuals belonging to *Ardea* spp. were included in the study, representing five of the 60 water birds examined. Although the detection of *G. duodenalis* was limited, with only two positive samples, this study provides, as far as we know, the first species-level confirmation of *Giardia* in wild birds in Greece, as previous investigations reported the parasite only at the genus level in free-ranging avian populations [6]. The lower positivity rate observed in our study (0.9%) compared to the previous report (6%) may be explained by key differences between the studies, including the type of clinical samples analyzed (cloacal swabs versus digestive tract samples), host species composition, and the condition of the birds examined (live versus dead individuals). Similar methodological and ecological factors may also account for the lower prevalence observed here, in relation to the average European prevalence of 13.7% [36]. The detection of *G. duodenalis* in ecologically distinct *Ardea* species is noteworthy, as, to our knowledge, this represents the first molecular identification of this *Giardia* species in these hosts [15]. Members of this bird genus are traditionally associated with the avian-specific parasite *Giardia ardeae* [15,36], so the presence of *G. duodenalis* is most plausibly interpreted as incidental spillover, reflecting environmental exposure rather than an established host–parasite relationship. Ecological traits of the host species support this interpretation. *A. ibis* frequently forages in terrestrial habitats, including pastures and agricultural landscapes near livestock, increasing potential contact with mammalian fecal contamination, while *A. cinerea*, more closely associated with freshwater habitats, may acquire the parasite via contaminated surface waters. These observations highlight the role of ecologically diverse heron species as sentinels of environmental contamination and underscore multiple pathways through which *G. duodenalis* may be introduced into avian hosts.

The presence of *Cryptosporidium* spp. in the present study was confined to two raptor species, *Buteo buteo* and *Asio otus*. Detection in *B. buteo* is consistent with earlier parasitological studies in Greece [6] and Spain [37], where this species has previously been reported as positive for *Cryptosporidium* spp. and *C. parvum*, respectively. By contrast, the detection of *Cryptosporidium* spp. in *A. otus* has not been previously documented and represents, based on the current knowledge, the first worldwide molecular evidence of this protozoan in the species, as earlier reports have only recorded *C. baileyi* in scops owls [13,38,39]. As with *Giardia,* the positivity rate in our study (0.9%) was lower than that (13%) reported in a previous similar investigation in Greece [6], and lower than that observed in other European countries [13,14,37,39], likely for the same reasons discussed above. The small number of positive findings in predatory birds suggests that exposure is likely linked to feeding ecology, such as ingestion of infected prey or indirect contact with contaminated environments, rather than to stable host–parasite associations. Overall, these findings broaden current knowledge of *Cryptosporidium* host range in wild raptors and underscore the value of targeted surveillance in understudied avian taxa. The two positive samples were further investigated for *C. hominis* and *C. parvum*, the primary species responsible for human infections, but both tested negative.

In the present study, none of the admitted birds tested positive for *Mycobacterium avium*. Although infected tissues remain the sample of choice for confirmation of avian mycobacterial infections by culture, microscopy, or molecular methods, PCR testing of cloacal swabs has been previously applied in live birds and represents a valid, non-invasive approach for detecting mycobacterial DNA when gastrointestinal shedding occurs [40]. Shedding of mycobacteria in feces and cloacal secretions is known to be intermittent and influenced by the stage and severity of infection; consequently, birds with subclinical or localized infections may not shed detectable quantities of organisms at the time of sampling [40]. Even under experimental conditions, PCR and culture of fecal material have demonstrated lower sensitivity compared with tissue-based diagnostics, particularly during early stages of infection or in the absence of systemic dissemination [41]. Therefore, the absence of *M. avium* detection in cloacal swabs in this study does not necessarily indicate a lack of exposure or infection at the population level, but may instead reflect a low prevalence of active intestinal shedding and the sporadic nature of mycobacterial excretion, especially given that the majority of sampled birds were admitted due to traumatic injuries rather than clinical disease. In similar studies conducted across European countries, the presence of *M. avium* in wild birds was also investigated along with other *Mycobacterium* species, but no positive results were obtained [42,43].

Since our initial PCR screening targeted *M. avium*, the main species responsible for avian tuberculosis in birds, yielded negative results, we expanded our molecular analyses to detect other mycobacterial species to better understand the diversity of mycobacterial exposure in the sampled population. Mycobacterial DNA was detected in six samples (Ct values ≤ 37), five in the category of raptors, and one in the category of water birds (Supplementary Tables S2 and S3), but the relatively low bacterial load precluded genotyping of the *Mycobacterium* species. These findings nevertheless indicate that the sampled birds were exposed to mycobacteria other than *M. avium*. This highlights both the circulation of non-avium mycobacteria and the intermittent shedding patterns, reinforcing the notion that cloacal swab PCR provides a useful, albeit limited, snapshot of mycobacterial exposure in live birds.

When viewed in the context of previously published European wildlife surveillance studies discussed above, the overall pathogen detection rate observed in our study (17.5%) appears broadly consistent with reported findings from other countries, although direct comparisons are inherently constrained by differences in sampling design, host composition, health status of examined birds, and diagnostic methodologies. These similarities support the role of Mediterranean wild bird populations as contributors to the broader European circulation of infectious agents detected in wild avifauna and underscore the importance of coordinated wildlife surveillance efforts across countries.

From a public health perspective, even moderate detection rates in free-living wild birds are epidemiologically meaningful, given their potential for environmental shedding and the diversity of ecological niches they occupy, some of which involve proximity to human-modified environments. In this context, as *C. psittaci* is estimated to account for approximately 1% of community-acquired pneumonia cases globally [19], its detection in 3.8% of sampled wild birds suggests that wild avifauna in Greece may represent a potential source of occupational and environmental human exposure. For the remaining pathogens detected here, sequencing was attempted, but did not yield sufficient data for reliable genotypic characterization. Consequently, their direct zoonotic relevance cannot be conclusively established. Further molecular investigation is therefore warranted to better clarify the public health significance of wildlife-associated pathogens.

## 5. Conclusions

Several studies have reported, worldwide, the presence of a wide range of infectious zoonotic agents in wildlife, denoting their role as important reservoirs and disseminators. However, there is limited, if any, data concerning our country for the prevalence of *Cryptococcus* spp., *Chlamydia psittaci*, *Giardia duodenalis*, and *Cryptosporidium* spp. in wild avian life. By using a non-invasive sampling method, in free-living wild birds, we showed the presence of these pathogens in specific categories of raptors, corvids, and water birds, from areas covering all parts of Greece. Notably, we provide evidence that specific categories are more prone to specific infectious agents, and certain parts of the country are burdened with higher pathogen loads than others. Even more importantly, our study provides, to the best of our awareness, the first worldwide molecular evidence of the presence of *G. duodenalis* in *Ardea species* and *Cryptosporidium* spp. in *A. otus*, and also reports *C. psittaci* in *Botaurus stellaris* and *Plegadis falcinellus*. Our findings will contribute to strengthening awareness and promoting sustained surveillance and monitoring of wildlife populations, thereby supporting the implementation of effective mitigation strategies.

## Figures and Tables

**Table 1:** List of primers and probes used for the molecular detection of pathogens.

Pathogen	Target Gene	Primer & Probe Sequence	Reference
*Cryptococcus* spp.	*18S rRNA*	5′ GTTGCAGTTAAAAAGCTCGT 3′ 5′ ACGGCGATCCTAGAAACC 3′ 5′ FAM-TGTGCAGGGGAACCAGGAAT-BHQ1 3′	[24]
*Chlamydia psittaci*	*incA*	5′ GCCATCATGCTTGTTTCGTTT 3′ 5′ CGGCGTGCCACTTGAGA 3′ 5′ FAM-TCATTGTCATTATGGTGATTCAGGA-MGB 3′	[25]
*Giardia duodenalis*	*Giardin*	5′ CGTGAAGATGATCAAGGAC 3′ 5′ CTGGTTCTGGAGATTTGTCT 3′ 5′ FAM-AGGAGATCGACACCATGGCTGC-BHQ1 3′	[26]
*Mycobacterium avium*	*ITS*	5′ GGTGGGGTGTGGTGTTTGAG 3′ 5′ CACGTCCTTCATCGGCTCTC 3′ 5′ FAM-GTGGCCGGCGTTCATCGAAATGTGT-BHQ1 3′	[27]
*Cryptosporidium* spp.	* 18S rRNA *	5′ CATGGATAACCGTGGTAACT 3′ 5′ TACCCTACCGTCTAAAGCTG 3′ 5′ FAM-CTAGAGCTAATACATGCGAAAAAA-MGB 3′	[26]

**Table 2:** The genera of the birds participated in this study, and their corresponding numbers and percentages per category.

Bird Category	Genus	Proportion of the Total Numbers per Category	Percentage
Raptors	*Buteo*	48/125	38.4%
	*Circus*	4/125	3.2%
	*Falco*	11/125	8.8%
	*Accipiter*	20/125	16.0%
	*Asio*	7/125	5.6%
	*Athene*	4/125	3.2%
	*Bubo*	12/125	9.6%
	*Circaetus*	3/125	2.4%
	*Otus*	11/125	8.8%
	*Pernis*	1/125	0.8%
	*Strix*	1/125	0.8%
	*Tyto*	3/125	2.4%
Corvids	*Corvus*	11/27	40.8%
	*Garrulus*	4/27	14.8%
	*Pica*	12/27	44.4%
Water birds	*Anas*	1/60	1.7%
	*Ardea*	5/60	8.3%
	*Botaurus*	7/60	11.7%
	*Ciconia*	3/60	5.0%
	*Egretta*	1/60	1.7%
	*Chroicocephalus*	4/60	6.6%
	*Larus*	34/60	56.6%
	*Pelecanus*	2/60	3.3%
	*Phalacrocorax*	1/60	1.7%
	*Phoenicopterus*	1/60	1.7%
	*Plegadis*	1/60	1.7%

**Table 3:** Bird population origin and their corresponding numbers and percentages per geographical unit.

Geographical Unit	Proportion of the Total Samples	Percentage
Eastern Macedonia and Thrace	8/212	3.8%
Central Macedonia	12/212	5.7%
Western Macedonia	4/212	1.9%
Epirus	4/212	1.9%
Thessaly	14/212	6.6%
Ionian Islands	12/212	5.7%
Western Greece	20/212	9.4%
Central Greece	21/212	9.9%
Peloponnese	15/212	7.0%
Attica	66/212	31.1%
North Aegean	8/212	3.8%
South Aegean	11/212	5.2%
Crete	17/212	8.0%

**Table 4:** Wild birds’ genera and species found positive in the pathogens tested, their corresponding numbers per pathogen, expressed as absolute numbers and percentages, and the total prevalence for each species.

					No of Positive Birds (%)				
Bird Group	Family	Genus/Species	Common Name	No. of Birds Sampled	*Cryptococcus* spp.	*Chlamydia psittaci*	*Giardia duodenalis*	*Cryptosporidium* spp.	Total
RAPTORS	*Accipitridae*	*Buteo*							
		*Buteo Buteo*	Common buzzard	46	7 (15.2)	-	-	1 (2.2)	8 (17.4)
		*Buteo rufinus*	Long-legged buzzard	2	-	-	-	-	-
		*Accipiter*							
		*Accipiter nisus*	Eurasian sparrowhawk	20	1 (5.0)	1 (5.0)	-	-	2 (10.0)
		*Pernis*							
		*Pernis apivorus*	European honey-buzzard	1	1 (100.0)	-	-	-	1 (100.0)
		*Circus*							
		*Circus pygargus*	Montagu’s harrier	1	-	-	-	-	-
		*Circus aeruginosus*	Western marsh harrier	3	-	-	-	-	-
		*Circaetus*							
		*Circaetus gallicus*	Short-toed snake eagle	3	3 (100.0)	-	-	-	3 (100.0)
	*Falconidae*	*Falco*							
		*Falco tinnunculus*	Eurasian kestrel	5	-	-	-	-	-
		*Falco peregrinus*	Peregrine falcon	4	-	-	-	-	-
		*Falco naumanni*	Lesser kestrel	2	-	-	-	-	-
	*Tytonidae*	*Tyto*							
		*Tyto alba*	Western barn owl	3	-	-	-	-	-
	*Strigidae*	*Asio*							
		*Asio otus*	Long-eared owl	7	1 (14.3)	-	-	1 (14.3)	2 (28.6)
		*Athene*							
		*Athene noctua*	Little owl	4	-	-	-	-	-
		*Otus*							
		*Otus scops*	Eurasian scops-owl	11	-	-	-	-	-
		*Bubo*							
		*Bubo bubo*	Eurasian eagle-owl	12	-	-	-	-	-
		*Strix*							
		*Strix aluco*	Tawny owl	1	1 (100.0)	-	-	-	1 (100.0)
Raptor subtotal				125	14 (11.2)	1 (0.8)	-	2 (1.6)	17 (13.6)
CORVIDS	*Corvidae*	*Corvus*							
		*Corvus cornix*	Hooded crow	8	1 (12.5)	1 (12.5)	-	-	2 (25.0)
		*Corvus corone*	Carrion crow	1	-	-	-	-	-
		*Corvus corax*	Common raven	1	-	-	-	-	-
		*Corvus frugilegus*	Rook	1	-	-	-	-	-
		*Pica*						-	
		*Pica Pica*	Eurasian magpie	12	-	4 (33.3)	-	-	4 (33.3)
		*Garrulus*							
		*Garrulus glandarius*	Eurasian jay	4	-	-	-	-	-
Corvid subtotal				27	1 (3.7)	5 (18.5)	-	-	6 (22.2)
WATER BIRDS	*Ardeidae*	*Ardea*							
		*Ardea cinerea*	Gray heron	3	-	-	1 (33.3)	-	1 (33.3)
		*Ardea ibis*	Western cattle-egret	1	-	-	1 (100.0)	-	1 (100.0)
		*Ardea purpurea*	Purple heron	1	-	-	-	-	-
		*Botaurus*							
		*Botaurus stellaris*	Eurasian bittern	1	-	1 (100.0)	-	-	1 (100.0)
		*Botaurus minutus*	Little bittern	6	1 (16.6)	-	-	-	1 (16.6)
		*Egretta*							
		*Egretta garzetta*	Little egret	1	-	-	-	-	-
	*Ciconiidae*	*Ciconia*							
		*Ciconia ciconia*	White stork	3	1 (33.3)	-	-	-	1 (33.3)
	*Anatidae*	*Anas*							
		*Anas platyrhynchos*	Mallard	1	-	-	-	-	-
	*Laridae*	*Larus*							
		*Larus michahellis*	Yellow-legged gull	34	5 (14.7)	-	-	-	5 (14.7)
		*Chroicocephalus*							
		*Chroicocephalus ridibundus*	Black-headed gull	4	-	-	-	-	-
	*Pelecanidae*	*Pelecanus*							
		*Pelecanus crispus*	Dalmatian pelican	2	2 (100.0)	-	-	-	2 (100.0)
	*Phalacrocoracidae*	*Phalacrocorax*							
		*Phalacrocorax carbo*	Great cormorant	1	-	-	-	-	-
	*Phoenicopteridae*	*Phoenicopterus*							
		*Phoenicopterus ruber*	American flamingo	1	1 (100.0)	-	-	-	1 (100.0)
	*Threskiornithidae*	*Plegadis*							
		*Plegadis falcinellus*	Glossy ibis	1	-	1 (100.0)	-	-	1 (100.0)
	Water bird subtotal			60	10 (16.7)	2 (3.3)	2 (3.3)	-	14 (23.3)
	Total			212	25 (11.8)	8 (3.8)	2 (0.9)	2 (0.9)	37 (17.5)

**Table 5:** Geographical distribution of the positive samples and the geographical regions’ contributing percentages to the total amount of the positive samples per pathogen.

	No of Positive Birds (%)			
Geographical Unit	*Cryptococcus* spp.	*C. psittaci*	*G. duodenalis*	*Cryptosporidium* spp.
Eastern Macedonia and Thrace	1 (4.0%)	-	-	-
Central Macedonia	4 (16.0%)	-	-	-
Western Macedonia	1 (4.0%)	-	-	-
Epirus	-	-	-	-
Thessaly	2 (8.0%)	-	-	-
Ionian Islands	1 (4.0%)	1 (12.5%)	1 (50.0%)	-
Western Greece	2 (8.0%)	-	-	-
Central Greece	3 (12.0%)	2 (25.0%)	-	-
Peloponnese	1 (4.0%)	-	-	1 (50.0%)
Attica	5 (20.0%)	3 (37.5%)	-	1 (50.0%)
North Aegean	2 (8.0%)	-	-	-
South Aegean	1 (4.0%)	1 (12.5%)	-	-
Crete	2 (8.0%)	1 (12.5%)	1 (50.0%)	-
Total	25	8	2	2

**Table 6:** Pathogens’ prevalence in different geographical areas across the country and proportions of the positive samples for each pathogen to the total numbers of samples from each geographical unit.

Geographical Unit	*Cryptococcus* spp.	*C. psittaci*	*G. duodenalis*	*Cryptosporidium* spp.	Total Prevalence
Eastern Macedonia and Thrace	1/8 (12.5%)	-	-	-	1/8 (12.5%)
Central Macedonia	4/12 (33.3%)	-	-	-	4/12 (33.3%)
Western Macedonia	1/4 (25.0%)	-	-	-	1/4 (25.0%)
Epirus	-	-	-	-	-
Thessaly	2/14 (14.3%)	-	-	-	2/14 (14.3%)
Ionian Islands	1/12 (8.3%)	1/12 (8.3%)	1/12 (8.3%)	-	3/12 (25.0%)
Western Greece	2/20 (10.0%)	-	-	-	2/20 (10.0%)
Central Greece	3/21 (14.3%)	2/21 (9.5%)	-	-	5/21 (23.8%)
Peloponnese	1/15 (6.7%)	-	-	1/15 (6.7%)	2/15 (13.4%)
Attica	5/66 (7.6%)	3/66 (4.6%)	-	1/66 (1.5%)	9/66 (13.7%)
North Aegean	2/8 (25.0%)	-	-	-	2/8 (25.0%)
South Aegean	1/11 (9.1%)	1/11 (9.1%)	-	-	2/11 (18.2%)
Crete	2/17 (11.8%)	1/17 (5.9%)	1/17 (5.9%)	-	4/17 (23.6%)

## Data Availability

The original contributions presented in this study are included in the article and Supplementary Material. Further inquiries can be directed to the corresponding authors.

## Supplementary Materials

The following supporting information can be downloaded at: https://www.mdpi.com/article/10.3390/pathogens15030308/s1, Supplementary Table S1: Level of Detection (LOD) of the qPCR assays used in this study, expressed as copies/mL (cp/mL); Supplementary Table S2: Primers and probe used for the molecular detection of *Mycobacterium* spp. Pathogens [44]; Supplementary Table S3: Wild birds’ genera and species found positive for *Mycobacterium* spp.
