# Supplementary material for: Molecular Evidence of Zoonotic Pathogens in Free-Living Wild Birds: A Greek Surveillance Study
Source: Pathogens. 2026 Mar 11;15(3):308. doi: 10.3390/pathogens15030308 (PMC13029732; doi:10.3390/pathogens15030308)
Supplement: Supplementary file 1 [file pathogens-15-00308-s001.zip › pathogens-4146407-supplementary.pdf]

Supplementary Table S1. Level of Detection (LOD) of the qPCR assays used in this study, expressed as copies/mL (cp/mL)

**Pathogen**	**Target Gene**	**LOD (cp/μL)**
Cryptococcus spp.	*18S rRNA*	0.036
Chlamydia psittaci	*incA*	0.9
Giardia duodenalis	*Giardin*	1.2
Mycobacterium avium	*ITS*	0.7
Cryptosporidium spp	*18S rRNA*	0.9

Supplementary Table S2. Primers and probe used for the molecular detection of *Mycobacterium* spp. pathogens.

**Pathogen**	**Target Gene**	**Primer & Probe Sequence**	**Reference**
Mycobacterium spp.	*ITS*	5′ GGGTGGGGTGTGGTGTTTGA 3′<br>5′ CAAGGCATCCACCATGCGC 3′<br>5′ FAM- TGGATAGTGGTTGCGAGCATC-TAMRA 3′	[44]

Supplementary Table S3. Wild birds′ genera and species found positive for *Mycobacterium* spp.

**Bird Category**	**Genus/Species**	**No of positive birds *Mycobacterium* spp.**
Raptors	*Buteo*<br>*Buteo buteo*	3
	Accipiter<br>*Accipiter nisus*	1
	Falco<br>*Falco naumanni*	1
Water birds	*Anas*<br>*Anas platyrhynchos*	1

Supplementary Reference

44. Angelakis E.; Roux V.; Raoult D.; Rolain J-M. Real-time PCR strategy and detection of bacterial agents of lymphadenitis. *Eur. J. Clin. Microbiol. Infect. Dis.* **2009,** *28,* 1363–1368. https;//doi.org/10.1007/s10096-009-0793-6.
